# Method for analyzing spike patterns with Markov transition matrices and Kullback-Leibler divergence

**DOI:** 10.1186/1471-2202-14-S1-P340

**Published:** 2013-07-08

**Authors:** Carl F Sabottke

**Affiliations:** 1 Department of Biological Sciences, Louisiana State University, Baton Rouge, Louisiana 70803, USA

## 

We describe a novel method for analyzing neural data that uses a combination of Markov transition matrices and Kullback-Leibler divergence to characterize spike history and spike patterns. For this method, the interspike intervals (ISIs) are divided into bins by quantiles, and a Markov transition matrix is computed for the ISI sequence. This Markov transition matrix is then compared to another Markov transition matrix constructed under the assumption of independent spiking. We then compute the Kullback-Leibler divergence between the distributions of ISI transition probabilities for each bin. The purpose of this method is to quantify the effects of spike history on neuron output and to help better characterize the flaws associated with assuming independent spiking.

We test this method on both simulated data and experimental data from primary visual cortex of cats, publicly available through CRCNS [1-3]. Interspike intervals are simulated based on exponential and gamma distributions fit to the corresponding experimental ISI distributions. Artificial data is also constructed by randomly reshuffling the temporal order of the experimental ISI sequences. Figure 1 shows a comparison between the experimental and simulated data for a typical neuron in the study. After comparison, we find that the Kullback-Leibler divergence for the experimental data is significantly greater than it is for the simulated data, but only for the bins at the extreme ends of the distribution. For spike time transitions after both the smallest and longest 10% of ISIs in the experimental data, the Kullback-Leibler divergence for these quantile groups is in many cases almost double the Kullback-Leibler divergence of the simulated data, and it can be more than an order of magnitude higher in some rarer cases. However, for transitions in the middle range, the Kullback-Leibler divergence is on average less than for the distribution tails, and the values for the experimental and numerical data are more similar.

**Figure 1:**
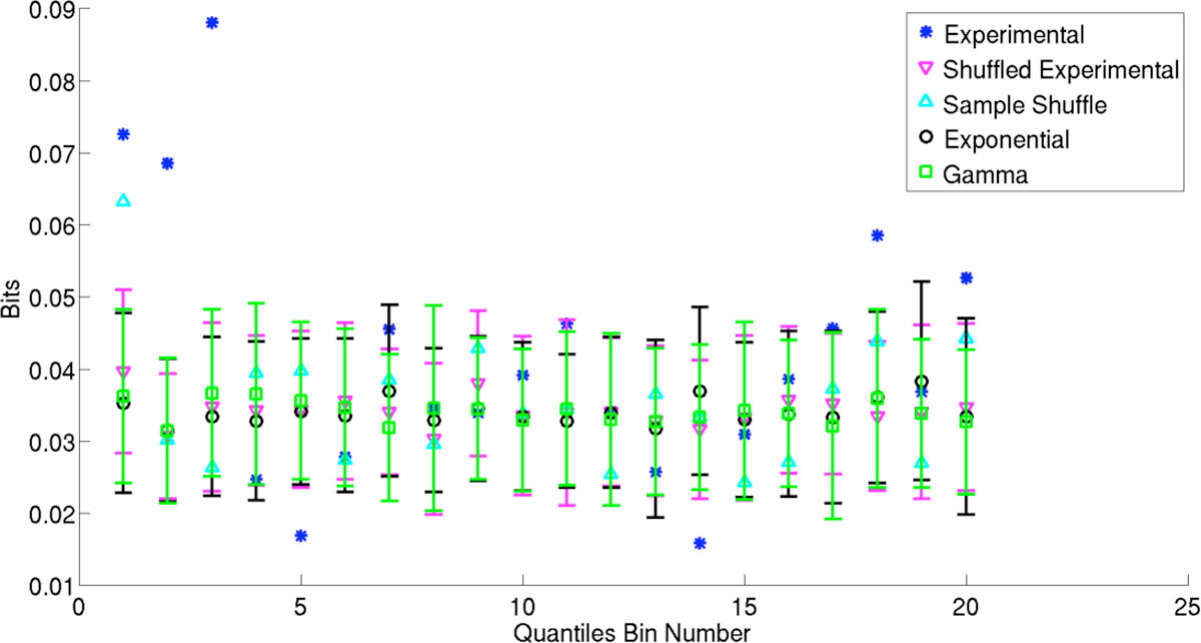
**Kullback-Leibler divergence estimates in bits for experimental and simulated ISI distributions broken up into 20 quantile bins**. Fifty spike trains of equal length were simulated for each category of artificial data. Averages and standard deviations for these simulated spike trains are shown for each quantile bin.

## Conclusions

These results emphasize that the main shortcoming of simple spiking models, like the Poisson model, is a failure to account for spike history and spike patterns related to bursting and long periods of silence. This also suggests that neural systems can gain an advantage in computational efficiency by accounting for spike timing aspects of bursting and extended periods of silence within the neural code.
